# Editorial: Human-Nature Interactions: Perspectives on Conceptual and Methodological Issues

**DOI:** 10.3389/fpsyg.2020.607888

**Published:** 2020-11-19

**Authors:** Tadhg E. MacIntyre, Juergen Beckmann, Giovanna Calogiuri, Aoife A. Donnell, Marc V. Jones, Christopher R. Madan, Mike Rogerson, Noel E. Brick, Mark Nieuwenhuijsen, Christopher James Gidlow

**Affiliations:** ^1^Health Research Institute, University of Limerick, Limerick, Ireland; ^2^Department of Sport and Health Science, Technical University of Munich, Munich, Germany; ^3^Science Centre of Health and Technology, University of South-Eastern Norway, Kongsberg, Norway; ^4^Faculty of Social and Health Sciences, Inland Norway University of Applied Sciences, Elverum, Norway; ^5^School of Food Science and Environmental Health, Technological University Dublin, Dublin, Ireland; ^6^Department of Psychology, Manchester Metropolitan University, Manchester, United Kingdom; ^7^Department of Psychology, University of Nottingham, Nottingham, United Kingdom; ^8^School of Sport, Rehabilitation and Exercise Sciences, University of Essex, Colchester, United Kingdom; ^9^Department of Psychology, Ulster University, Coleraine, United Kingdom; ^10^Barcelona Institute of Global Health, Barcelona, Spain; ^11^Staffordshire University, Stoke-on-Trent, United Kingdom

**Keywords:** ecosystem services, biodiversity, health, virtual reality, urbanization, nature based interventions, mental health, nature based solutions

Urban agglomerations expose citizens to ever-increasing risks from heat, air pollution, noise stress, and reduced nature connectedness. Concurrently, accumulating evidence suggests various health benefits by exposure to urban natural spaces (World Health Organization, 2016a; Bratman et al., 2019). Existing research suggests an array of benefits of contact with nature which are linked to physical activity (e.g., green exercise), active travel, and residential proximity to greenspace. Psychological benefits appear to be related to mood, well-being, attention and pro-environmental behavior; physiological benefits have been described in terms of increased physical activity, improved cardiovascular parameters, reduced stress hormones, and enhanced immune resources (Bowler et al., 2010; Li, 2010; Park et al., 2010; Calogiuri and Chroni, 2014; Hartig et al., 2014; van den Bosch and Sang, 2017).

Nature offers a low-cost non-invasive solution for mental health and well-being with the potential to reduce inequities. This has never been so relevant. COVID-19 related restrictions on mobility and associated reduced (or lack of) access to many recreational venues, has meant that engaging with nature, by visiting nearby natural environments (Samuelsson et al., 2020) or through home gardening (Walljasper and Polansek, 2020) has been an important means of staying active and managing stress—in some case also to mitigate food insecurity. Technology, especially emerging technologies such as virtual reality (VR), can also facilitate human-(virtual) nature interactions when contact with real nature is not possible (Litleskare et al., 2020).

Environmental psychology has helped us to understand human-nature interactions from a transactional perspective (Gifford, 2013). Ecosystems services have been applied to explain the benefits and risks of such interactions (Bratman et al., 2019). More recently, *nature-based solutions* (NBS) have come to the fore supported by the EU Biodiversity strategy 2030, UN Global Compact and the IUCN global standard for NBS. There is a growing scientific imperative in achieving consensus on the optimum measures, methodological approaches, theoretical frameworks, and concepts to enhance our understanding of human-nature interactions (Frantzeskaki, 2019). The race for upscaling and proliferation of NBS has commenced and yet the speed of advancement of conceptual understanding and methodological rigor lags behind. Despite more than three decades of research since the advent of the *biophilia* hypothesis, researchers' conclusions have been limited by methodological challenges. Few studies have employed measures that are directly comparable with national or international surveys (e.g., WHO-5). Theoretical assumptions from environmental psychology have not been readily supported by models based on biological plausibility or neural implementation. A range of methodological approaches in the assessment of predisposing factors including nature connectedness and prior experience has limited the capacity of systematic reviews to conduct reliable comparisons (Lahart et al., 2019). Standardization of measures and conceptual clarity among researchers would facilitate more robust research, cross-cultural comparisons and provide clearer evidence for the future decisions on investment in nature-based solutions with the capacity to address many societal challenges.

In launching this Research Topic, our objective was to capture contemporary perspectives on the conceptualization and measurement of human-nature interactions, and advance future research perspectives. The ubiquitous nature of the challenge is exemplified by a diverse and expansive list of countries of our contributors, which ranges among 15 different countries including Australia, Brazil, Denmark, Finland, Germany, Ireland, Norway, Peru, Portugal, Singapore, South Africa, Spain, Switzerland, the UK, and the USA. Twenty articles were included in the collection. These included an array of approaches, with nine original research articles, two brief research report articles, four perspective articles, two reviews, and three systematic reviews. Many provide novel viewpoints in our understanding of human-nature interactions, in relation to both, the effects of being in contact with nature and potential underlying mechanisms explaining the relationship. More specifically, the articles included in this collection have investigated the extent to which exposure to nature can affect indices of physical and mental health (Berry et al.; Gritzka et al.; Mygind et al.), psychophysiological parameters (Becker et al.; Browning, Mimnaugh et al.; Hunter et al.; Litleskare and Calogiuri; Reeves et al.), cognitive restoration (Olszewska-Guizzo et al.; Stevenson et al.), and environmental attitudes and behaviors (Rosa and Collado). Two articles evaluated “best-dose” of nature exposure, i.e., the most effective amount of time required to obtain health benefits (Hunter et al.; Meredith et al.).

Several of the included articles have investigated or discussed possible explanations for the health and restorative benefits of interacting with nature. These included studies on brain activity associated with perception of natural environments (Mahamane et al.; Olszewska-Guizzo et al.; Reeves et al.), the impact of scene oscillations on psychological responses to exposure to virtual nature (Litleskare and Calogiuri), and how eye movements contribute to explain restorative processes (Stevenson et al.). Two studies have investigated the impact of being exposed to natural environments on health-related behaviors that may, in turn, contribute explaining the health effects of interacting with nature; these included physical activity (Becker et al.) and healthy decision-making (Berry et al.). One article proposed a theoretical framework that can be adopted to conceptualize the complex human-nature interaction (Brymer et al.). Two articles examined the relationship between the concepts of nature connectedness and social relational values (Kleespies and Dierkes) or between the concepts of nature connectedness and emotions (Petersen et al.), whereas Render et al. explored the association between individuals' personality and their choice of work environment. Other topics included related to challenges encountered by interdisciplinary research groups (Berry et al.), the conditions of captive amphibians (Measey et al.), and the concept of place identity (Peng et al.) and vulnerability (Tallman et al.).

This Research Topic highlighted the growing interest in studying nature effects on cognition through use of cutting-edge technologies and instruments, such as virtual reality (VR) and measurements of brain activity. This is encouraging, as the hope is that using more immersive, yet experimentally controlled, exposure to natural environments increasing the precision that will allow comparison of different environment exposures (e.g., different types of nature, built environments). Moreover, modern cognitive neuroscience approaches such as electroencephalography (EEG) and functional magnetic resonance imaging (fMRI) may be more useful in indicating the mechanism underlying these nature benefits, by indicating how different brain regions are engaged between the different experimental conditions (e.g., see Madan et al., 2019, for further background). In the present Research Topic, several studies were based on this approach, using VR or 3D imaging to expose participants to different environments (Browning, Mimnaugh et al.; Litleskare and Calogiuri; Olszewska-Guizzo et al.), and/or performed objective measurements such as EEG assessments of brain activity (Mahamane et al.; Olszewska-Guizzo et al.; Reeves et al.), biomarkers of stress (Becker et al.; Hunter et al.; Reeves et al.), and assessments by mobile eye-tracker (Stevenson et al.). Innovative instruments and methodologies were also represented, with one study involving a novel approach to implement “self-managed” nature experiences interventions in the context of daily life (Hunter et al.) and another examining the effectiveness of using a novel low-cost wearable technology to conduct in-loco assessments of brain activity and biomarkers of stress (Reeves et al.).

The rationale for this Research Topic was to advance the methodological rigor in the field and the research appears to have supported the need for such an approach. For example, quality of the evidence was often deemed low in both systematic reviews (Gritzka et al.; Mygind et al.) and this had recently been reported in the broader literature (Lahart et al., 2019). This limiting factor inhibited both analyses and effect sizes were thus not calculated in either study. With this in mind and the aforementioned discourse, we present our recommendations for future research.

## Recommendations

On the base of wider literature and emerging knowledge in this field, as well as the new knowledge generated through the articles included in this Research Topic, we highlight the following recommendations for future research:

- *Nature experiences during COVID19 pandemic—*The COVID19 global pandemic brought to the fore the need to increase both the access and availability of nature in urban areas for multifunctional inter-generational social, physical, and mental health (Nieuwenhuijsen, 2020). One learning point for the field is that the transactional viewpoint of ecosystem services (for health) does not adequately address the complex interactions between humans and nature. Investment in nature-based solutions by the EU, for example, highlights that transdisciplinary approaches more readily capture for the potential reciprocal benefits. Within the Research Topic a wealth of theoretical approaches had been applied (e.g., ecological systems) which pivoted beyond the traditional dichotomous approach of Stress Reduction Theory and Attention Restoration Theory. As the published papers in this Research Topic have demonstrated, theory-driven research using diverse explanatory frameworks are recommended to enable the research of today to resonate far into the future.
- *Nature Exposure and Experience—*A parsimonious approach focused on dose-response effects may overlook the role of the participants' attention or mindset during nature exposure. To this end, Bratman et al. (2019) refer to the nature experience as comprising both “dose” and “interaction”; i.e., the specific ways in which people interact with nature may account for differential impacts of nature exposure on health-related outcomes. Nature connectedness is a key variable that requires further insight-do we need an urban nature connectedness construct? Furthermore, there is a need to account for other factors including natural environment quality (a potential factor of inequality, World Health Organization, 2016b), the attention of the participant and their perception of the setting.
- *Immersive technology to enhance methodological rigor—*Use of immersive technology such as virtual reality (VR) and, especially, immersive-virtual environments (IVE) offers great opportunities for conduction experiments in highly controlled conditions. While we encourage researchers to make use of this technology in experimental design to enhance methodological rigor, we also warn about challenges associated with this technology. While VR and IVE technology can provide more vivid experiences of nature as compared to non-immersive virtual exposure (e.g., videos or pictures), recent analyses show that exposure to virtual nature provides psychological responses to a lesser extent than real nature (Browning et al., 2020). This needs to be taken into account when interpreting findings of experiments using environmental exposure via VR or IVE. At the same time, we encourage more studies that aim to understand how to improve the quality of virtual nature experiences.
- *Technological nature to promote and augment human-nature interactions—*Recently attention has been drawn to the role technological nature (especially in form VR, augmented reality, and mobile applications) in promoting and augmenting human-nature interactions, particularly among groups of individuals with limited access to real nature (Litleskare et al., 2020). Studies in this field are extremely scarce, thus we encourage researchers to explore the effectiveness of different approaches as well as their underlying mechanisms.
- *Multidimensional health—*An array of methods have been employed in an attempt to comprehensively account for the possible positive and negative impacts of human nature interactions. Subjective scales, objective markers (e.g., EEG), and biomarkers (e.g., cortisol) can provide converging evidence for the impact and will potentially expand the range of factors to be considered in the future. For instance, the construct of psychological resilience, has rarely been subject to study by researchers in this field despite the obvious overlap with the concept of resilience in natural systems. A broad view of health could also enable greater generalizability across settings from the workplace to the classroom, to the urban and rural communities supported by a broad consensus or standardization in the measures of the common constructs and outcomes.
- *Interdisciplinary and Transdisciplinary—*The evidence presented in this Research Topic and our recommendations are not specific to one field or discipline but have implications across the broader field beyond environmental psychology. Environmental psychology, after four decades could benefit from a reset on the approaches required to address the ever-pressing wicked problems of climate change, biodiversity deficit, environmental degradation, rapid urbanization, and global pandemics.
- Our actions, decisions and omissions are so closely intertwined with ecological effects that they can hardly be considered separately (Stokols, 2018). Nature provides a potential low stigma and low risk intervention, and the benefits for human and environmental health are potentially reciprocal. These complex inter-relationships requires theory driven questions, sophisticated methods and complex analyses. By advancing the concepts and methods a window of opportunity opens for human nature interactions to be more clearly elucidated.

## Author Contributions

All authors listed have made a substantial, direct and intellectual contribution to the work, and approved it for publication.

## Conflict of Interest

The authors declare that the research was conducted in the absence of any commercial or financial relationships that could be construed as a potential conflict of interest.
